# Persistent Asthma in Childhood

**DOI:** 10.3390/children9060820

**Published:** 2022-06-01

**Authors:** Cilla Söderhäll, Ann-Marie Malby Schoos

**Affiliations:** 1 Department of Women’s and Children’s Health, Karolinska Institutet, SE-17177 Stockholm, Sweden; 2 Astrid Lindgren’s Children’s Hospital, Karolinska University Hospital, SE-17164 Stockholm, Sweden; 3 COPSAC, Copenhagen Prospective Studies on Asthma in Childhood, Herlev and Gentofte Hospital, University of Copenhagen, 2820 Gentofte, Denmark; 4 Department of Pediatrics, Slagelse Hospital, 4200 Slagelse, Denmark

Asthma is the most common chronic disease in children and a cause of great distress for both the children and their families. Various overlapping phenotypes exist [1], complicating aligned research in the field and meaningful, personalized treatment of the disease. Young children are particularly diverse with numerous and variable phenotypic presentations in early life that correspond to different outcomes. Despite the high reported rates of remission of asthma, the disease is usually considered treatable but not curable once present. Understanding the determinants that affect the course of diagnosed asthma, e.g., the avoidance of environmental or occupational exposures, is therefore important for tertiary prevention, since asthma persistence is associated with frequent and severe symptoms and with the development of impaired lung function [2]. Asthma treatment guidelines have proven useful in standard care and the reduction of adverse outcomes in patients with asthma; however, the phenotypic heterogeneity within the disorder indicates a need for personalized medicine as opposed to a one-size-fits-all treatment approach. A possibility to predict disease course (transient vs. persistent) would be a very valuable tool when deciding on the treatment course and intensity in a particular child.

In the paper by Holmdahl et al. [3], a cohort of preschool wheezers were followed until school-age in an attempt to identify risk factors for persistent asthma. In this high-risk cohort, more than 70% of the 113 children had an asthma diagnosis at the age of 7, with rhinovirus-induced wheeze in early life as the most associated risk factor, which confirms findings from previous studies. The majority of the children had recurrent wheeze already at inclusion, and more than 80% were hospitalized due to an acute episode of wheeze at the time point of inclusion, indicating severe symptoms. Children that developed asthma were also found to be more severely affected the first year after inclusion with a need for more hospital care due to respiratory symptoms in relation to preschool wheezers that did not develop asthma. Their results support that early viral-induced recurrent wheeze and more severe symptoms at preschool age are associated with persistent asthma.

In the paper by Lovrić et al. [4], a machine learning approach was applied to try and predict treatment outcomes in 355 pediatric asthma patients (aged 2–17) and ten adolescents (aged 18–22) and further identify the key variables relevant to the underlying mechanisms of asthma. During the study, the patients were treated with inhaled corticosteroids (ICS) (alone or in combination with long-acting beta2-agonists) and/or leukotriene receptor antagonists. They found that spirometry results (forced expiratory volume in 1 second (FEV1) and maximal expiratory flow at 50% (MEF50)) did not perform well and had a lower predictive quality of treatment success. However, fractional exhaled nitric oxide (FeNO) performed better suggesting that it can be used as a predictor of steroid responsiveness more consistently than spirometry, which aligns well with FeNO being a good biomarker of the Th2-related allergic inflammatory response, as interleukin-13 promotes nitric oxide (NO) synthase activity and NO production. Finally, the level of asthma control (LOAC) assessed by a pediatric pulmonologist reflected treatment success best, highlighting the importance of subjective symptoms expressed by the children and their families when evaluating treatment effects in pediatric asthma. In addition, MEF50-based outcomes were related to high-sensitivity C-reactive protein (hsCRP) and performed similarly to FEV1 when predicting treatment response, highlighting the importance of the distal airways in children with asthma. As the peripheral airways are the predominant site of airway inflammation, they could very well also be an important site of airflow obstruction in asthmatic children and thereby involved in the pathophysiology and resistance to treatment with ICS. The authors further suggest that distal airway impairment may be present despite rare and mild asthma symptoms and normal FEV1 suggesting that MEF50 should be considered as a variable in the diagnostic process of childhood asthma.

In the paper by Berghea et al. [5], the efficacy of treatment with the monoclonal anti-IgE antibody, Omalizumab, was tested in 12 Romanian children with severe persistent allergic asthma aged 6–18 years. During the 1-year treatment, they observed a decrease in severe exacerbations, FEV1, MEF50, eosinophil count, and ICS use. Further, there were no adverse reactions recorded that resulted in the discontinuation of treatment. This is the first study to report treatment outcomes of Omalizumab in a pediatric Romanian population. Although many studies have shown great promise in treatment with biologics in severe asthma [6], the long-term effects remain to be elucidated, including whether or not they have an effect on asthma persistency.

This Special Issue focuses on persistent, severe asthma covering risk factors, suggested monitoring and mechanisms of treatment outcomes, and finally treatment of severe, persistent allergic asthma.

## References

[1] Just J., Bourgoin-Heck M., Amat F. (2017). Clinical phenotypes in asthma during childhood. Clin. Exp. Allergy.

[2] McGeachie M.J., Yates K.P., Zhou X., Guo F., Sternberg A.L., Van Natta M.L., Wise R.A., Szefler S.J., Sharma S., Kho A.T. (2016). Patterns of Growth and Decline in Lung Function in Persistent Childhood Asthma. N. Engl. J. Med..

[3] Holmdahl I., Filiou A., Stenberg Hammar K., Asarnoj A., Borres M.P., van Hage M., Hedlin G., Söderhäll C., Konradsen J.R. (2021). Early Life Wheeze and Risk Factors for Asthma—A Revisit at Age 7 in the GEWAC-Cohort. Children.

[4] Lovrić M., Banić I., Lacić E., Pavlović K., Kern R., Turkalj M. (2021). Predicting Treatment Outcomes Using Explainable Machine Learning in Children with Asthma. Children.

[5] Berghea E.C., Balgradean M., Pavelescu C., Cirstoveanu C.G., Toma C.L., Ionescu M.D., Bumbacea R.S. (2021). Clinical Experience with Anti-IgE Monoclonal Antibody (Omalizumab) in Pediatric Severe Allergic Asthma—A Romanian Perspective. Children.

[6] Agache I., Akdis C.A., Akdis M., Canonica G.W., Casale T., Chivato T., Corren J., Chu D.K., Del Giacco S., Eiwegger T. (2021). EAACI Biologicals Guidelines-Recommendations for severe asthma. Allergy.

